# World Athletics regulations unfairly affect female athletes with differences in sex development

**DOI:** 10.1080/00948705.2024.2316294

**Published:** 2024-03-05

**Authors:** Hilary Bowman-Smart, Julian Savulescu, Michele O’Connell, Andrew Sinclair

**Affiliations:** a Murdoch Children’s Research Institute, Melbourne, Australia; b Department of Paediatrics, University of Melbourne, Melbourne, Australia; c Australian Centre for Precision Health, University of South Australia, Adelaide, Australia; d Oxford Uehiro Centre for Practical Ethics and Wellcome Centre for Ethics and Humanities, University of Oxford, Oxford, UK; e Centre for Biomedical Ethics, National University of Singapore, Singapore; f Endocrinology and Diabetes, Royal Children’s Hospital, Melbourne, Australia

**Keywords:** Sport, female athletes, differences in sex development, 5-ARD, Caster Semenya

## Abstract

World Athletics have introduced regulations preventing female athletes with certain differences in sex development from competing in the female category. We argue these regulations are not justified and should be removed. Firstly, we examine the reasoning and evidence underlying the position that these athletes have a substantial mean difference in performance from other female athletes such that it constitutes an advantage, and argue it is not sufficient. Secondly, if an advantage does exist, it needs to be demonstrated it is unfair. We argue the advantage would not be unfair because to say otherwise relies on a presupposition about whether these athletes are female, which involves contradictory and inconsistent definitions of sex. Thirdly, we contend that even if it is established that there is an advantage and it is unfair, the response of requiring athletes to take testosterone-suppressing medication is not appropriate and is unfair.

In elite athletics, it is widely considered critical to maintain the ‘spirit of sport’, an idea which is simultaneously fundamental and difficult to define (Pugh and Pugh 2021). Important principles relevant to the spirit of sport include justice, equality and fairness. However, the ambiguity in how we understand and apply these concepts has led to significant debates and disputes that have been escalated to the European Court of Human Rights.

A central issue is the right to compete in the female category for female athletes with certain differences in sex development (DSD).^1^ Attempts to regulate the female category have been occurring since the 1930s (Schultz 2021). However, recent regulations have further pressed the issue. The International Association of Athletics Federations (IAAF), now known as World Athletics (WA), has repeatedly tried to introduce measures to prevent female athletes with hyperandrogenism (higher levels of androgens, such as testosterone) from competing in their natural state, and they appear to have succeeded.

These attempts to prevent participation of female athletes with a DSD have been subject to appeals to the Court of Arbitration in Sport (CAS). One such appeal was made by Caster Semenya, a South African middle-distance runner. Semenya is a two-time Olympic gold medallist in the 800 m. She has had her right to participate in the female category of elite athletics questioned since 2009, when WA requested a sex verification test. In 2019, Semenya lost her appeal at CAS and the regulations were upheld (Reuters 2020). Upon a series of further appeals and escalations, the European Court of Human Rights found in July 2023 that Semenya’s right to non-discrimination had been violated by the Swiss state (Drywood 2023). This does not mean that the regulations will be overturned and Semenya will be able to return to competition, but it does demonstrate the negative impact these regulations can have on athletes and sporting competitions. In a press release after the ruling, WA stated that the current DSD regulations, as approved in March 2023, will remain in place but that they will liaise with the Swiss Government for the next steps (World Athletics 2023b).

We do not believe these regulations restricting the participation of female athletes with a DSD are evidence-based, ethically justified, or an appropriate response. There are not good reasons to support them. Although the Swiss federal court has supported the CAS decision, we argue they should not stand. In this paper, we will touch on central points in the debate around athletes with a DSD in elite sports, and add to the philosophical literature around the conceptual coherence and justification for categorisation in sports.

We will firstly examine the evidence for whether there is a substantial mean difference in performance between female athletes with a DSD and those without (an advantage). Secondly, we examine whether this substantial mean difference in performance (if it exists) is conceptually and philosophically relevant to the process of categorising elite athletes, and thus whether it is an unfair advantage. Finally, we examine whether the requirements of the WA are an appropriate response to an unfair advantage. We conclude by arguing these regulations fail to be supported on all three of these counts.

## The WA regulations – increasing in strictness but not in clarity

In the past decade, multiple sporting bodies have enacted policies to address the question of criteria for sports that have different categories based on sex. A particular focus has been the inclusion of athletes – particularly female athletes – with a DSD. The term DSD (also known as intersex variations, or more exact aetiological terminologies) refers to a heterogeneous group of congenital conditions that arise when chromosomal, gonadal, or anatomical sex is atypical (see Table 1).

In 2023, the International Olympic Committee (IOC) released a position statement outlining a non-binding framework on fairness, inclusion and non-discrimination on the basis of gender identity and sex variations, outlining ten relevant principles (Martowicz et al. 2023). One such principle was ‘no presumption of advantage’, which was then the focus of a critique in a subsequent joint position statement by the International Federation of Sports Medicine (FIMS) and the European Federation of Sports Medicine Associations (EFSMA) (Pigozzi et al. 2022). The regulations put in place by WA have been central to the debate. Here, we outline the change in the WA regulations over time, their rationale and evidence base, and criticise the increasingly stringent but arbitrary and unreasonable thresholds athletes are required to meet in order to compete in the female category.

Semenya’s wins early in her career have been a flashpoint for these efforts to regulate sex categorisation. In 2011, WA attempted to limit participation of hyperandrogenic females in elite athletics, by limiting the allowable level of testosterone for female athletes to 10 nmol/L (International Association of Athletics 2011). However, WA were forced to suspend these regulations in 2015 following an appeal by Dutee Chand, an Indian sprinter with hyperandrogenism (Pape 2019). The CAS ruled there was insufficient evidence to support these rules, and WA had 2 years to gather enough supporting evidence (Pape 2019).

In this time, WA changed tack in their approach to regulation. Instead of a testosterone limit for female athletes generally, they introduced new regulations limited to specific kinds of female athletes (some females with a DSD), and specific events – e.g. track events between 400 m and a mile. During this process, WA further restricted the regulations to only cover specific DSDs. The female athletes covered by these regulations are generally females with a 46,XY karyotype (see Table 2) as well as some other common DSDs (Court of Arbitration for Sport 2019). Although presence of a Y chromosome is typically associated with a male appearing phenotype, this is not universally the case. Variants in genes involved in gonadal development (e.g. *SRY* (Berta et al. 1990; Sinclair et al. 1990), steroid hormone biosynthesis (e.g. 5α-reductase deficiency) or androgen responsiveness give rise to various forms of 46,XY DSD that may result in a female appearing, or predominantly female appearing, phenotype (Berta et al. 1990; O’Connell et al. 2021). In children with these variations, circulating androgen levels after puberty may be higher than in 46,XX females; however their relative androgen exposure is typically significantly lower than that of 46,XY males (see Table 1).

In the original 2018 submission to the CAS, although much of it is redacted, there is a strong focus on the condition 5α-Reductase deficiency (5-ARD) in relation to Semenya (Court of Arbitration for Sport 2018). 5-ARD is caused by a gene variant on chromosome 2, resulting in a lack of production of the 5α-reductase enzyme. This enzyme converts testosterone into dihydrotestosterone (DHT), a much more potent androgen. The presence of DHT *in utero* is necessary for the development of typical male appearing genitalia (Kumar and Barboza-Meca 2020), hence someone with 5-ARD often develops an atypical genital phenotype. When *in utero* DHT production is markedly reduced, the phallus may be considered typical of a clitoris, while appearance in others may be ambiguous (resembling either an enlarged clitoris or a smaller than typical penis). Testes are present but may not be externally palpable or recognised. Assignment to female sex of rearing is common, with virilisation occuring at puberty. A very similar clinical picture (female genital appearance at birth, but higher levels of androgen production from puberty) ensues with 17β-hydroxysteroid dehydrogenase type 3 (17β-HSD3).

Another common cause of a female appearing phenotype in individuals with an XY karyotype is androgen insensitivity syndrome (AIS) (Hughes et al. 2012). In AIS, the androgen receptors do not function typically, or at all. Therefore, even though testosterone is produced (and may appear high in blood tests), the body cannot respond to it in the usual way; it may have no effect (complete AIS – phenotype is entirely typically female appearing) or some effect (partial AIS – phenotype varies). Athletes with complete AIS are excluded from the regulations as they are not ‘Relevant Athletes’, but athletes with partial AIS may be covered (see Table 2). Ovotesticular DSD is a rare DSD where a combination of testicular and ovarian tissue develops. The clinical phenotype varies, depending on the amount of functioning gonadal tissue present.

In addition to restricting the regulations to athletes with particular DSDs, WA have significantly lowered the threshold for blood testosterone level over the years. The testosterone limit in force from November 2019 for female athletes with a DSD – at 5nmol/L – was lower than that in 2011 (10nmol/L). These regulations were further updated in March 2023 to the even lower limit of 2.5 nmol/L (World Athletics 2023a). Furthermore, while in the 2019 regulations the athletes were only required to reduce their blood testosterone level for a continuous period of at least six months, the updated 2023 regulations state that the athlete must have continuously maintained the serum testosterone concentration <2.5 nmol/L for a period of at least 24 months – a significant increase.

The evidence underlying these decisions is unclear. The justification for lowering the serum testosterone concentration even lower in the 2023 regulations made reference to a ‘survey’ (World Athletics 2023a, note 3). This survey was itself a review published by (Handelsman, Hirschberg, and Bermon 2018). In this review of the medical and scientific literature – although not systematic ‘ the authors took the position that the eligibility criterion should be <5nmol/L in order make ’an allowance for women with mild hyperandrogenism, notably with polycystic ovary syndrome [PCOS] … ‘ (Handelsman, Hirschberg, and Bermon 2018). However, the WA appears to have drawn on a particular finding from this review, which is that women without DSDs have a much lower mean serum testosterone concentration (95% confidence interval: 0.06 and 1.68 nmol/L) than women with DSDs (World Athletics 2023a, note 1). Although this review also highlighted the higher level in women with PCOS, WA explicitly states that the DSD regulations would not apply to female athletes with PCOS.

The regulations are also inconsistent, with some components being highly specific without sufficient justification or explanation as to why, and others not specific at all. As part of the regulations, the WA notes (World Athletics 2023a, notes 4 and 5) that ‘due to circadian fluctuations in the blood levels of testosterone, the blood sample(s) should be collected between 8 am and 10 am’ (time zone not specified; presumably local), ‘with the athlete not having taken part in any strenuous physical exercise for at least two hours before the time of blood collection’ (World Athletics 2023a, 23). Therefore – more precisely – a female athlete with a DSD must have a testosterone serum concentration of <2.5nmol/L between the hours of 8 am and 10 am after resting for two hours. This specification seems relatively arbitrary. While the regulations aim to account for fluctuations over the course of a day, there appears to be no specification as to point during the month or year, despite the evidence across sexes for the fluctuation of hormones such as testosterone over time periods – for example, the period of a menstrual cycle, or even potentially seasonally over the course of a year (Zornitzki et al 2022 Stanton, O’Dhaniel, and Huettel 2011). The application of justifying evidence throughout these regulations is inconsistent.

A further key concern with these regulations is with regards to criterion (3.1.3) in the regulations (see Table 2). This means the athlete must not have ‘sufficient androgen sensitivity’ to have a ‘material androgenising effect’. It is unclear how WA defines these terms, although they describe three levels of assessment, increasing in level of detail. The regulations state that a Level 1 assessment will use a combination of clinical examination and laboratory investigations, including blood and urine tests to begin with. The aim of this assessment is to confirm the blood testosterone level; gather information to assist in diagnosis; and gather information ‘to assist in assessing whether the athlete is androgen insensitive (and, if so, to what degree)’ (World Athletics 2023a, Appendix 2, note 2, 17). To do so, ‘[t]he examining physician will assess the athlete in particular for clinical features associated with pronounced and chronic cases of female hyperandrogenism.’ (World Athletics 2023a, Appendix 2, note 7, 18); however, what constitutes ‘material androgenising effect’ is not defined. Arguably, sensitivity may vary in different tissues. A Level 2 Assessment involves the review of an ‘Expert Panel’, and a Level 3 Assessment may involve genetic testing, imaging, and psychological assessment to establish a diagnosis (World Athletics 2023a, Appendix 2, 19-20).

Of note, there is no universally accepted or standardised means of assessing androgen sensitivity, hence this criterion is open to significant inter-assessor variability. Tannenbaum and Bekker have previously raised the point that there are no ‘reproducible, valid laboratory tests’ to detect androgen sensitivity, and that assessments are based on factors such as clitoral size which are open to false interpretation (Tannenbaum and Bekker 2019, 1). This has in fact moved WA from a specific definition based on chromosomal karyotype to one which is vague and centred around a poorly-defined concept of what an androgenising effect is. This means that two athletes with partial AIS could potentially be treated differently based on characteristics (e. g. clitoral size) that would not otherwise be considered relevant if they had a 46,XX karyotype. It is not clear if, how, or what factors would be considered under the WA regulations.

In terms of guidance for such assessments, the 2023 regulations simply state that WA ‘may provide a checklist to assist in the collection of all potentially relevant information’ (World Athletics 2023a, Appendix 2, note 7, 18). The ‘degree of the athlete’s androgen insensitivity (if any)’ appears to be fundamentally at the discretion of the Expert Panel (World Athletics 2023a, Appendix 2, note 17, 20). The Expert Panel who will be drawn from ‘a pool of independent medical experts’ appointed by WA’s CEO, ‘from which a suitably qualified panel of experts (the “Expert Panel”) may be formed … ‘ (World Athletics 2023a, section 4.1, 7). It is not stated what type of medical experts, how they will be assessed in terms of their suitability, or how the panel will be chosen from the larger pool appointed by the WA’s CEO.

They also state that the benefit of any doubt will favour the athlete - but it is difficult to imagine a case in which there is no doubt. If we consider two potentially ‘relevant athletes’ - females with partial androgen insensitivity syndrome (PAIS) and females with ovotesticular DSD - we can see how further complexities can arise. The former athlete may have high testosterone levels but reduced sensitivity (PAIS) while the latter may have a testosterone level just above the threshold of 2.5 nmol/L but typical androgen sensitivity (ovotesticular DSD). Although the overall effective androgen exposure arising from these scenarios could be similar, only the female athletes with PAIS may be allowed to compete. It is difficult to see how this is fair. These regulations appear to be attempting to be thorough to address the range of possible cases that may be addressed, but simultaneously fail to define or explain key terms or steps used in assessing these athletes – such as ‘material androgenising effect’.

The rationale for these regulations is that the higher level of testosterone produced by some female athletes with a DSD results in a substantial mean difference in performance such that it impacts the ability of WA to ‘give equal opportunities to all athletes to participate … and to provide them with **fair** and **meaningful** competition conditions … ’; with this being important in order to motivate athletes to make ‘the huge commitment and sacrifice required to excel in the sport’ and thus inspire new generations of athletes to do so in turn (World Athletics 2023a, section 1.1.1, 2)

Furthermore, the regulations also assume that requiring the relevant athletes to take medication to reduce their testosterone levels is the appropriate way to mitigate this difference in performance. Embedded in this are several assumptions and/or arguments: that elevated level of testosterone in female athletes with relevant DSDs results in increased performance compared to female athletes without; that this difference in performance is unfair in some way; that certain medications can ameliorate this difference in performance; and, because the difference in performance is unfair, requiring athletes to take them is warranted. We examine these points and find them to be lacking.

### Is there a performance difference?

We first must establish whether there is a substantial difference in performance between female athletes with a DSD covered by the WA regulations and those without, such that it confers an advantage of some degree. Furthermore, we need to be able to quantify the difference in performance we can attribute specifically to the fact that the athlete has a DSD. If we are going to use this as a basis for disqualifying athletes, then we need to be confident in the science.

The evidence does not appear to warrant this level of confidence. The 2021 FIMS statement on this topic states that ‘current physiological data are insufficient to adequately inform policy’ (Hamilton et al. 2021, 1409). They do take the position that serum testosterone concentration remains the most useful, albeit imperfect, biomarker (Hamilton et al. 2021, 1407). The studies on which the WA drew heavily on for their 2019 regulations (Bermon 2017; Bermon et al. 2018) were conducted by WA-affiliated scientists, and the data had also not been fully released for verification by researchers not affiliated with WA (Pielke, Tucker, and Boye 2019). Pielke *et al* have called for retraction of both studies on scientific grounds, as they found several errors in the incomplete dataset provided to them and could not replicate the results (Pielke, Tucker, and Boye 2019). Sőnksen *et al* have similarly presented concerns with some of the statistical analysis used (Sőnksen et al. 2018), as have Menier *et al* (Menier 2018) and Franklin *et al* (Franklin, Betancurt, and Camporesi 2018). Indeed, and importantly, Bermon *et al* have issued a correction stating that their research cannot support a causal inference (Bermon and Garnier 2021).

Following on from these studies, some members of the same research group who produced the Bermon *et al* papers were involved in a blinded, placebo-controlled study to assess the effects of exogenous testosterone (applied as a cream) on physical performance in healthy young females. They found that increased serum testosterone levels were associated with increased physical performance (time to exhaustion) and lean body mass in the testosterone-supplemented group (Hirschberg et al. 2020). However, females with a DSD are not taking exogenous testosterone, they are producing endogenous testosterone. Furthermore, depending on the nature of their DSD, they have differing levels of sensitivity to that testosterone, or differing levels of ability to convert it to more potent androgens such as DHT. Conversely to the above study, a 2021 analysis of an Australian dataset of 716 pre-menopausal females found that total endogenous testosterone was not associated with lean mass index or handgrip strength (Alexander et al. 2021). Another study by Eklund *et al* found that higher levels of DHT, although not serum testosterone itself, was associated with higher performance (Eklund et al. 2017). As previously noted, one of the key features of 5-ARD is that it impacts the conversion of testosterone into DHT. This suggests a more complex picture than is captured by the WA regulations. Even if we accept that testosterone can be causally associated with a difference in performance, what these studies have provided evidence for is an average competitive advantage of testosterone in some events, by relatively small margins (e.g. a margin of approximately 1.78% in the 800m (Bermon and Garnier 2017)) in females *without a DSD*. That is not to say that a <2% difference means little when on the racetrack – in elite sports, it can mean everything – but the margin demonstrated is far below the difference in performance of men, on average, compared to females, which is between 10 and 12% higher performance (Handelsman 2017). Only nine of the over 2,000 participants in the Bermon *et al* study were identified as females *with* a DSD. They did not demarcate females with high levels of androgens by cause (e.g. polycystic ovarian syndrome, or PCOS). It may well be that this testosterone advantage only applies to females *without* a DSD or females who do not have higher endogenous androgen production.

Another point raised in favour of supporting these regulations is that when on testosterone-suppressing medication such as supra-physiological doses of oestrogen, these female athletes’ performances decrease, and thus this is evidence testosterone impacts performance. Bermon *et al* cite a 5.7% decrease in performance in females with a DSD resulting in hyperandrogenism when testosterone levels are decreased (Bermon 2017). WA has argued the review by Handelsman *et al* (2018) suggests that the reduction in performance is due to the suppression of testosterone. However, other evidence also exists that challenges this. A study by Crewther *et al* (2018) found that oral contraceptives in elite female athletes (who did not have a DSD) did decrease testosterone levels, but did not affect performance (Crewther et al. 2018). A 2020 systematic review found a slight negative impact of oral contraceptives on performance in females who would otherwise naturally menstruate, but trivial group-level effects (Elliott-Sale et al. 2020).

Furthermore, we should consider that if oral contraception reduces performance, we must examine whether this is attributable to testosterone reduction or some other cause. For example, Semenya attributed reduction in performance to side-effects of the testosterone-reducing medication such as weight gain, nausea and fevers (Court of Arbitration for Sport 2018). For these reasons, when further discussing a possible performance gap, we will not use figures that are drawn from *decreases* in performance while on testosterone suppressing medication.

There are three elements to consider – the *strength* of the evidence, the *degree* of the mean difference in performance (if any) demonstrated by the evidence, and then the *cause* of the mean difference in performance. We do not believe it has satisfactorily been established that females with a ‘relevant DSD’ have a substantial mean difference in performance over all other females, due to the increased level of testosterone, to a degree that aligns with the differences underlying the current rationale for our existing categories (males and females). It is therefore not appropriate for regulations to be based on this assumption. Indeed, the WA regulations suggest that athletes with a DSD should be given the benefit of the doubt in cases of uncertainty.

### If there is an advantage, is it unfair?

However, let us imagine it has been established that there is a substantial difference in performance, such that it constitutes an advantage. This alone is not enough. Different kinds of athletes have all kinds of advantages over others. Thus, it is not sufficient to exclude athletes from a category solely on the basis that they are faster or stronger than other athletes in that category. What needs to be established is that the advantage is relevant to how the categories are decided and defined. So, although we do not believe it has been satisfactorily established, we now move forward assuming Semenya has a mean difference in performance of approximately 2% over other female athletes. We argue that this advantage would not prevent ‘fair and meaningful competition conditions’ – that is, it would not be *unfair*.

Let us examine why WA believes this advantage to be unfair such that it warrants exclusion of female athletes with a DSD. There is clearly a careful emphasis on how WA is defining the criteria for the female *category*, rather than defining what a ‘female’ is. Sebastian Coe, president of WA, has said ’ … we need to be clear about the competition criteria for these two categories [males and females]’ (IAAF Athletics 2018). The recent March 2023 regulations, when referring to the conditions on participation, state that they are ‘[i] n no way are they intended as any kind of judgement on or questioning of the sex or the gender identity of any athlete’ (World Athletics 2023a, section 1.1.5, 2). However, given that the female category is, by basic definition, for female athletes, it is exceedingly difficult to see how criteria can be applied to determine whether an individual athlete is eligible for the female category, without making some comment on whether that athlete *is* female.

Nonetheless, WA argues that this ruling is not a statement on the sex or gender of any individual athlete, but instead is ‘ … about levelling the playing field to ensure fair and meaningful competition in the sport of athletics where success is determined by talent, dedication and hard work rather than other contributing factors’ (IAAF Athletics 2018). However, how can we separate out talent from natural abilities – natural abilities that Semenya has? Why are Semenya’s natural abilities classed as ‘other contributing factors’ rather than seen as a contribution to her natural physical talent, in the same way a highly flexible person has a natural talent as a gymnast? How do we determine which ‘other contributing factors’ result in an unfair or unmeaningful competition? And why is elevated testosterone an ‘other contributing factor’ in 5-ARD but not in hyperandrogenism from CAH? The obvious difficulty is that athletes inevitably have performance advantages for a range of other reasons, including physical factors such as height, socioeconomic status, region of birth, and level of parental involvement. Many of these are difficult or impossible to modify or account for when developing a categorisation system. A key question is then why it is necessary to regulate testosterone to ensure a level playing field amongst female athletes, and not these other factors (Jennings and Braun 2023). Given the focus on defining the criteria for the female category, these regulations are inevitably making a statement about whether Semenya is female, but it appears that WA are loathe to be perceived as doing just that. Indeed, two Advocates of the High Court of South Africa noted this ‘internal contradiction’ within the WA regulations – simultaneously saying they accept these athletes as female, while excluding them from the female category and offering the option of competing in the male category (Court of Arbitration for Sport 2018).

Before proceeding to consider whether this advantage would be unfair, we must look at what it means for an advantage to be unfair. The WA approach of focusing on a ‘level playing field’ is unhelpful given the wide range of advantages athletes can have, as described above, and thus we must look at other definitions. Camporesi and Hämäläinen (2021) outline a view that an ‘unfair advantage’ would be an advantage that is not available to others within the same category (‘category attainability’). This does not mean that the advantage needs to be available to *everyone* within the same category; if, for example, 10% of females can attain the advantage, then category attainability applies and the advantage is not unfair. This is because if a strict attainability criterion is applied – whether a specific individual can obtain the advantage – then the number of categories we would need to use in sport would explode (Camporesi and Hämäläinen 2021). It is a possibility to do so – such as how boxing has weight classes – but the current question at hand is the eligibility for the ‘female’ and ‘male’ categories, not different categories or sub-categories within those categories. Moving forward with the category attainability approach to fairness, the key question becomes – as Camporesi and Hämäläinen note – how to define and justify the categories we use, in this case male and female.

### Defining female

Broadly – and very simplistically speaking, as will be touched on – sex refers to biology and gender refers to social role or self-identification. In sport, sex was assessed anatomically in the 1960s, and then by biological tests such as the presence of a Barr body (found in cells with multiple X chromosomes, e.g. a 46,XX karyotype) or the *SRY* gene (Reeser 2005). Due to controversies around discrimination and stigmatisation, and concerns about the reliability of genetic testing methods, WA dropped blanket sex testing in 1992. The International Olympic Committee eventually followed suit by the time of the 2000 Sydney Olympics (Elsas et al. 2000). However, this does not mean that sex testing was abandoned entirely; individual athletes may still be subject to challenge (Heggie 2010).

Biological sex itself is not simple, with chromosomal, gonadal, or secondary sex characteristics all candidate definitions that would include or exclude different groups. For example, XY denotes the sex chromosomes that generally result in a male appearing phenotype. However, using the sex chromosomes for sex determination would run into some difficulties. For example, translocation of the *SRY* gene onto the X chromosome can result in 46,XX males with DSD. These males have XX (‘female’) chromosomes but the presence of the *SRY* gene causes testes to develop resulting in the male appearing phenotype (Wang et al. 2009). Similarly, variants in the *SRY* gene in 46,XY females with DSD have a ‘male’ chromosome set but a non-functioning *SRY* gene, so testes do not develop and a female appearing phenotype ensues (Berta et al. 1990). There are also cultural, social and racial dimensions to defining sex in sport and the categorisation of secondary sex characteristics; Schultz discusses how the dominance of African-American and Soviet women in the mid-20^th^ century fuelled anxieties about men infiltrating women’s sport (Schultz 2011). Given the impact of social context, it is particularly important not to rely on subjective assessments of whether or not particular features are considered ‘masculine’ or ‘feminine’. However, the WA regulations fail to engage with these complexities. As previously discussed, they rely on terms such as ‘material androgenising effect’, which is not clearly defined or testable, and is instead left to subjective assessment of unspecified ‘medical experts’ chosen by WA.

The way sex has been defined in the relevant research on athletics presumes certain kinds of classification. For example, as Pape and Pielke point out, a review attempting to show that the testosterone ranges for males and females do not overlap from the start explicitly separates females with a DSD from females without a DSD (Pape and Pielke 2019). Thus, those data showing testosterone level for ‘females’ already, by definition, excludes females with a DSD that produce high levels of androgens. Therefore, the data used to justify the separation of females with a DSD from females without a DSD presume this separation in the first place; Pape and Pielke describe this as ‘methodological circularity’ (Pape and Pielke 2019).

Caster Semenya is legally female, was assigned female at birth, raised as female, and identifies as female. It seems clear on the face of it, therefore, that Semenya is a female. Therefore, the view that Semenya’s advantage is unfair carries the inference that Semenya is really a male competing in the female category. This is illustrated by articles in the media which repeatedly refer to Semenya as a 46,XY *male* with a DSD (Coleman 2019).

Female athletes belong in the female category. Caster Semenya is a female. Therefore, Caster Semenya belongs in the female category. This is quite a straightforward point. However, if we accept that Semenya has an advantage over other female athletes *and* that this should disqualify her, while simultaneously accepting that she *is* female, or at least making no comment on her sex or gender, as WA is very keen to say, then the ruling is essentially saying that only certain *kinds* of female athletes belong in the female category. We now examine if this is justified.

### Other biological advantages are not seen as unfair – what is the difference?

Many athletes have biological advantages that give them an edge. The Finnish skier Eero Mäntyranta had a genetic variant that boosted the oxygen-carrying capacity of his red blood cells by 25–50%, winning several Olympic medals with this natural form of performance enhancement (McCrory 2003). He was not required to reduce his biological levels by donating blood. Many elite athletes are, essentially, freaks of nature. This is not to be disparaging, but simply to point out that many elite athletes possess physical traits well outside the normal range. For example, Michael Phelps has an unusually wide wingspan and very large feet. This results in a clear advantage for swimming, but this advantage is not seen as unfair.

This comparison between Semenya’s case and other biologically advantaged athletes is not new (Camporesi 2020; Cooper 2010). Semenya’s performances have been described as ‘so dominant’ that they ‘verge on mockery’ (Silkstone 2009). However, other athletes have dominated their sports; Usain Bolt, the 100m world record holder, has been described by an article on the official Olympics website as ‘the undisputed dominant force in recent times’ in sprinting (Nag 2020). Therefore, there must be some additional concern that differentiates Semenya’s case from these other exceptional athletes.

Indeed, there is a specific difference between factors such as Phelp’s wide wingspan and Semenya’s testosterone levels, which is the nature of the advantage. That is, we do not categorise swimming heats by wingspan, but we do categorise them by male or female. We now return to Camporesi and Hämäläinen’s view of unfairness as category attainability (Camporesi and Hämäläinen 2021). The question is not just about the existence and degree of the advantage – Phelps undeniably has a significant natural advantage – it is about the nature of the advantage, and its relationship with the logic of categorisation. Parry and Martínková (2021) demarcate ‘competition advantages’ (such as height) with ‘category advantages’, which are connected with the definition of the category itself.

The critical question then is *why* we make decisions about categories in sport. As two of the authors here have argued elsewhere, the rules and parameters of sport are not fixed and are subject to ongoing negotiation, based on a wide range of considerations (Bowman-Smart et al. 2021). One position is that sporting categories function to ‘protect’ certain groups of athletes who are disadvantaged and would otherwise have very low chances of success (Martínková 2020). We categorise athletic events by male/female because female athletes are disadvantaged compared to male athletes, and without such categorisation, there would be undesirable consequences – for example, limited opportunities for female athletes in elite athletics. The importance of having women represented on the podium is inextricably connected with the broader societal context and history of gender inequality. For example, the IOC framework on fairness, inclusion and non-discrimination explicitly recognises this, stating that it ‘ … acknowledges the significance of fair competition opportunities for the women’s category, given the historical and contemporary struggle for gender equality in sport’ (Martowicz et al. 2023, 26–27). In this case, the idea of categorising sport based on *other* physical metrics (e.g. height or weight) to be ‘fair’ is not necessarily useful, because the aim is not to facilitate participation or representation of *any* athlete with decreased performance relative to others. It is to facilitate representation of a class of athletes based on a particular kind of characteristic (i.e. sex), who would otherwise not be represented at the level of elite sport.

That is, if we categorise ‘males’ and ‘females’ as two groups, the performance difference between groups is only relevant such that it warrants separating these out as categories due to other objectives (e.g. ensuring representation, opportunity, etc). If there were no substantial difference in performance, and there are no other reasons to make these distinctions (e.g. religious beliefs), it is unlikely we would need to categorise sport this way. This is supported by the fact that some elite sports where there is no difference in performance between groups – such as dressage – have no categorisation based on sex. On the other hand, some ‘mindsports’ such as chess do have separate categories for women. This is often justified as a means of addressing the underrepresentation of women at the elite level for reasons other than performance differences (such as a hostile culture of the sport), but this idea has also been subject to significant critique (Punch et al. 2023).

If the key aim of categorisation is, for example, to ensure opportunity for and representation of *female* athletes, then there would be no need to object to the inclusion of athletes with a DSD unless you did not view them as female. If we take the category attainability view, it is impossible to make a statement about whether Semenya’s advantage is ‘unfair’ without the prior view that she does not belong in the female category. This is because if the advantage is attainable to those within the category (and Semenya is within the category), then the advantage is not unfair. Thus, while WA argues that their regulations are necessary to maintain fairness, it does not make sense to talk about whether it is necessary to redefine categories to ensure fairness. This is going backwards. It is the very definition of categories that *makes* something fair or unfair. It is not possible, as WA have, to argue that it is unfair for Semenya to compete in the female category without making a statement on whether or not Semenya herself is female.

It is true that Semenya’s testosterone levels are outside the typical range for a female. However, the WA ruling specifically applies to female athletes with specific DSDs (and, previously, specific events). The rule does not apply to female athletes *in general*. If there is an 46,XX female with CAH – a condition in 46,XX females that results in hyperandrogenism – then she is allowed to compete. There is no restriction on her level of testosterone, even if it is similarly elevated. This is because WA’s regulations have placed a focus on disordered *gonadal* steroidogenesis (see Table 2), and not *adrenal* steroidogenesis, as though the physical and biological source of the increased androgens, and furthermore the alleged advantage, is somehow conceptually and morally relevant. The implication is that females with XX chromosomes are ‘real’ females, and thus any advantage they have due to natural variation in androgen levels is fair.

Given that these regulations focus on females with altered gonadal ster-oidogenesis and explicitly exclude adrenal steroidogenesis again suggests it is not, as WA claim, purely about advantage. It is about advantage a *female* athlete should not rightly have claim to. If this debate were solely about ensuring a ‘level playing field’ – and not just about who deserves to compete as a *female* – then the testosterone limit would apply to all female athletes.

If there were a 46,XX female athlete who had the same times as Semenya, and had a non-gonadal variation that conferred the same advantage, she would be allowed to compete unhindered in the female category. That might be, for example, a genetic variant that greatly increases the production of red blood cells or eliminated lactic acid build-up – or an extraordinary sensitivity to normal levels of androgens. But she is allowed to compete and Semenya is not, even if the outcome is the same.

### New WA regulations acknowledge previous inconsistencies

The WA had previously ruled that because high-testosterone female athletes outperform low-testosterone female athletes in some events (e.g. the 800m), this is an unfair advantage and thus they should be disqualified from competing. However, the same exact data on which the 2019 regulations were based also has suggested that there are events where having *low* testosterone appears to confer an advantage, such as the 100m (Pielke, Tucker, and Boye 2019). Nonetheless, regulations were not made to artificially elevate testosterone levels to compete in these events. Furthermore, the Bermon *et al* paper also highlighted the hammer throw and pole vault as events where higher testosterone was associated with increased performance (Bermon and Garnier 2017). In fact, the advantage seen in these events (4.53% and 2.94%, respectively) is much higher than the advantage seen in the events they chose for the regulations. However, the 2019 regulations did not affect these events. Allegedly, the reasoning for this was that there was at the time no evidence that athletes with a DSD competing in these events (Court of Arbitration for Sport 2018). However, as Vilain and Martínez-Patiño note, this created an ‘absurd sex-shifting situation’ where an athlete would qualify as a female in one event, but not in another (Vilain and Martinez-Patiño 2019). The 2021 statement from the British Association of Sport and Exercise Science (BASES) describes both the relatively weak evidence on which this decision was based, as well as the ‘paradoxical’ application of the regulations to five middle-distance running events only (Herbert et al. 2021).

In response to these criticisms, the March 2023 version of the WA regulations expands them to cover all events. For ‘transitional cases’ – athletes who have already been competing in other events in the female classification – the period for assessment is shortened from 24 months to 6 months (World Athletics 2023a, 14). This demonstrates that WA *is* able to respond to inconsistencies where they are raised in the literature and broader debate, and we believe they must continue to address the further inconsistencies highlighted here.

### If it is an unfair advantage, is this the appropriate response?

If we accept there is an advantage, and this advantage is unfair, we then need to examine what is an appropriate response. We do not believe requiring athletes like Semenya to undergo a medical intervention to artificially reduce their testosterone to be able to compete is the appropriate response.

### Negative, disproportionate and unjust impacts on athletes with a DSD

The decision is unjust, unfair, and has negative and disproportionate impacts on athletes with a DSD, in several ways. Semenya has entered, trained, and competed fairly under the rules. To change them has undermined her capacity to compete, work and live, after a lifetime of investment. Even if the regulations stand – and while we do not believe this is sufficient – there should at the very least be a ‘grandmother clause’ for current athletes like Semenya or else they are unfairly burdened by the bungles of WA.

This ruling further discriminates and disadvantages athletes with a DSD to benefit those who do not have a DSD. Justice, particularly from the prioritarian view, involves giving priority to those are most disadvantaged, marginalised and worst off (Holtug 2017). Individuals with DSDs already experience stigma, discrimination and social disadvantage in many domains, while some will have significant additional medical considerations (e.g. sub- or infertility). To participate now as a female athlete at the elite level, you may participate so long as no ‘relevant information derived from a reliable source’ suggests that you have a DSD (World Athletics 2023a, section 4.4, 7), in which case your categorisation must be investigated and your eligibility determined. There are significant concerns about what would be considered ‘relevant information’ or a ‘reliable source’; the vagueness of these terms increases the opportunities for bad-faith actors to impact the likelihood of an athlete’s participation. This is likely to disproportionately impact not just athletes with a DSD, but any athlete who is gender non-conforming, as well as introducing further pressure on all female athletes to be gender-conforming and appear ‘feminine’ (Karkazis et al. 2012).

Furthermore, it sets back acceptance of people with a DSD and serves to perpetuate misunderstandings and further stigmatise and marginalise them more broadly (Carpenter 2020). At a societal level, sex variations are a poorly understood part of human diversity – greater awareness, understanding and inclusiveness on society’s part should be the goal. However, this ruling sends the opposite message. Although WA have repeatedly and carefully stated that this ruling is not a comment on Semenya’s status as a female, and that the regulations are not intended as a judgement on the sex or gender identity of any athlete, it is, as we have argued, difficult to interpret it any other way. Furthermore, public discourse and discussion about athletes with a DSD have frequently identified or equated them with transgender athletes. Indeed, the 2021 FIMS Consensus Statement – which primarily ends with a call for more scientific data – addresses transgender athletes and athletes with a DSD together (Hamilton et al. 2021). While this is also an important and relevant matter that connects into the broader debate around categories in sport, Semenya has stated that she herself is not transgender (Gregory 2023). There are related ethical issues and the arguments presented herein may have implications for this connected debate, but it is also important not to perpetuate misconceptions about what it means to have a DSD. As much as WA protests that they are not intending to pass judgement on sex or gender identity, they clearly and demonstrably are, and they have perpetuated a harmful discourse around people with DSDs. WA is not responsible for the media, but as we have noted, Semenya has also been repeatedly referred to as a male in coverage surrounding these regulations. She has herself stated that being referred to as a man was disrespectful to her and the ‘language they used to describe me was a humiliation’ (Gregory 2023).

### Medical intervention as disproportionate, unreasonable and unimplementable

Reducing or suppressing testosterone involves risk. The general method of testosterone reduction touted as being suitable is the use of hormonal medications such as high dose oestrogen or progesterone. However, even though such medications are widely used in the general population, they involve risks such as increased risk of blood clots, and the lowest possible doses to achieve the desired therapeutic effect should be used. However, athletes may need higher doses to achieve ‘effective’ suppression. When someone chooses to take this medication to prevent pregnancy or regulate their menstrual cycle, they are deciding based on the balance of benefits and harms for themselves. However, in Semenya’s case, these interventions interfere with a functioning body for highly uncertain benefits to *other* people, without any direct therapeutic effect.

We believe this is disproportionate and unreasonable. ‘Medical’ interventions to decrease the functioning of an individual – as opposed to improve it – is contentious enough. When this reduction in functioning is not even for the benefit of that specific individual, it becomes highly ethically suspect. Requiring someone to undergo a medical intervention may violate their human right to health (Mphidi and Lubaale 2020). As Semenya herself has said ‘They want me to take my own system down. I’m not sick. I don’t need drugs’ and ‘[The pills] make you sick. They take the soul out of you … it was dark’ (Brenner 2021; Gregory 2023).

If Caster Semenya is required to take medication to reduce her testosterone levels, this amounts to requiring her to experience risks for it to be a ‘fair’ competition. It is somewhat ironic that much of the well justified concern about performance-enhancing drugs is that their widespread use may effectively ‘force’ people to take them to compete, forcing athletes to experience increased risk. This rationale – that an environment that forces athletes to take drugs to compete is a bad one – is seemingly discarded in Semenya’s case. As Holmen et al (2022) argue, if medical interventions in the name of creating fairness in the sense of a ‘level playing field’ are justified, then this is in fact also a justification to allow performance-enhancing drugs.

Being an elite athlete is accompanied by risk already – a broken bone, a torn ligament, a concussion. However, we should not expect them to shoulder additional risk caused by unnecessary medical interventions. Indeed, we specifically take measures to prevent this. However, in this case, Caster Semenya is being forced to undertake the risk associated with this intervention to be able to compete.

Furthermore, we must question the ethics of a doctor who chooses to actively administer an intervention such as testosterone-lowering medication that risks harming the patient – in addition to side-effects, also if we conceive of lowering functioning as harm – for no benefit to the patient, and instead to benefit others. The World Medical Association has advised doctors not to administer testosterone lowering interventions, describing the regulation as ‘contrary to international medical ethics and human rights standards’ (World Medical Association 2019). Their use would be ‘off label’ and is for purposes other than the athlete’s health. The rules involve ‘strict liability’ which means the athlete is responsible for any failure to comply, even if unintentional and outside of the athlete’s control. Questions have been raised about whether it would even be legal for a doctor to administer this medication (McQuoid-Mason 2019). In response to the World Medical Association, the FIMS 2021 Consensus Statement contends that ‘this argument is an outdated approach’ and that such an intervention would be acceptable if the athlete is fully informed and not coerced (Hamilton et al. 2021, 1408). They also state that ‘Any treatment is a purely personal and private decision and no sports body should provide recommendations on treatment’ (Hamilton et al. 2021, 1402). However, this fails to engage with the dubious nature of ‘informed consent’ in a situation where eligibility to compete is dependent on agreeing to the intervention (Karkazis and Carpenter 2018). It also appears to implicitly position testosterone suppression as a form of ‘treatment’ rather than a harmful intervention an athlete must agree to in order to qualify to compete.

## Conclusion

It is important that sport is welcoming of all kinds of athletes. WA’s regulations unfairly limit a certain class of athletes from participating fully in this key domain of life. We do not believe these restrictions are justifiable. There are good reasons against them. These are not merely scientific questions; they are also ethical questions. It has not been satisfactorily demonstrated that female athletes with a DSD, such as Semenya, have a substantial advantage over other female athletes due to their DSD. If it is demonstrated they have an advantage, we do not believe this advantage would be unfair. Finally, even for those who think this advantage would be unfair, we do not think the requirement of testosterone-suppressing medication is the appropriate response. These regulations fail on all these counts. To make sport truly fair they should be repealed.

## Figures and Tables

**Table 1:** Differences in sex development relevant to the debate.

DSD	Karyotype	Impact of variation	Common clinical features	T >5 nmol/l in adulthood?	Impacted by regulations
5α-reductase type 2 deficiency	46, XY	Inability to convert Testosterone to dihydrotestosterone (DHT)	Atypical or female appearing genitalia at birth; androgen production increases from puberty	Likely	Yes
Partial androgen insensitivity syndrome	46, XY	Testes produce androgen but tissues have reduced sensitivity to androgen effect	Atypical genitalia at birth; variable subsequent androgen effect	Likely, but variable effect	Possibly*
17β-hydroxysteroid dehydrogenase type 3 (17β-HSD3) deficiency	46, XY	Inability to convert androstenedione to testosterone	Atypical or female appearing genitalia at birth; androgen production increases from puberty	Likely	Yes
Ovotesticular DSD	46,XX, 46, XY or 45X/ 46XY mosaicism	Atypical gonadal development; both ovarian and testicular tissue present	Atypical genitalia at birth. Variable androgen production thereafter#	Possible#	Yes
Congenital adrenal hyperplasia (CAH) (e.g. 21-hydroxylase deficiency)	46, XX	Adrenal androgen excess	Atypical genitalia, clitoromegaly at birth. Androgen excess reduces with usual glucocorticoid therapy.	Unlikely but possible^$^	No - explicitly excluded^^^

* if deemed to have ‘sufficient androgen sensitivity’ to have ‘material effect’; however, no validated / standardised test of receptor sensitivity

# Androgen production depends on degree of functioning testicular tissue in a given individual

$ Typically not, but may occur if glucocorticoid adrenal hormone replacement therapy is insufficient

^ Rules apply only to gonadal androgen excess (hence 46, XY DSD); basis for this is unclear. Explicitly excluded in endnote 6 of WA regulations (World Athletics 2023a, p.23)

**Table 2:** The criteria for ‘relevant athletes’ covered by the WA regulations (World Athletics 2023a, 5-6; World Athletics 2019, 4).

3.1 A ‘Relevant Athlete’ is an Athlete who meets each of the following three criteria:
3.1.1 They* have one of the following DSDs:
3.1.1.1 5α-reductase type 2 deficiency;
3.1.1.2 partial androgen insensitivity syndrome (aka PAIS);
3.1.1.3 17β-hydroxysteroid dehydrogenase type 3 (17β-HSD3) deficiency;
3.1.1.4 ovotesticular DSD; or
3.1.1.5 any other genetic disorder involving disordered gonadal steroidogenesis; and
3.1.2 as a result, they have a concentration of testosterone of 2.5 nmol/L or more in their serum**; and
3.1.3 they have sufficient androgen sensitivity for that** testosterone to have a material androgenising effect.	“An athlete with CAIS is not a Relevant Athlete. An athlete with PAIS will only be a Relevant Athlete if they are sufficiently androgen-sensitive for her elevated testosterone levels to have a material androgenising effect. The benefit of any doubt on this issue will be resolved in favour of the athlete.” This is similar to 2019 wording, except for pronouns (World Athletics 2019, note 4)	In end note 6 (p.23) they state:	“These DSD Regulations do not apply to any other conditions (including, without limitation, polycystic ovary syndrome and Congenital Adrenal Hyperplasia), even if such conditions cause the athlete to have testosterone levels in her blood above the normal female range.”

* pronoun changed from “she” in the 2019 regulations to “they” in the 2023 regulations

** previous wording in 2019 regulations: “as a result, she has circulating testosterone levels in blood of five (5) nmol/L or above”

*** wording changed from “those levels of testosterone” in 2019 regulations to “that testosterone” in 2023 regulations

## Footnotes

1 We note that there is some debate about the most appropriate language to use in this context.
